# A phase I study of nedaplatin, pemetrexed and thoracic intensity-modulated radiotherapy for inoperable stage III lung adenocarcinoma

**DOI:** 10.1186/s12885-016-2800-5

**Published:** 2016-10-07

**Authors:** Yiyu Lu, Weiguang Gu, Jin Deng, Hua Yang, Wen Yang

**Affiliations:** 1 Department of Oncology, Nanhai Hospital of Southern Medical University, Foshan, 528200 China; 2 Department of Radiotherapy, Cancer Center of Guangzhou Medical University, Guangzhou, 510000 China

**Keywords:** Lung adenocarcinoma, Chemotherapy, Nedaplatin, Pemetrexed, Intensity-modulated radiotherapy

## Abstract

**Background:**

Concurrent chemotherapy and radiation is the standard treatment for unresectable stage III Lung adenocarcinoma. However, no optimal concurrent chemotherapeutic regimen has been described. This study aimed to assess concurrent pemetrexed, nedaplatin and thoracic intensity-modulated radiotherapy followed by consolidation pemetrexed/nedaplatin for unresectable Stage IIIA/B lung adenocarcinoma.

**Methods:**

Patients with unresectable stage III lung adenocarcinoma received thoracic intensity-modulated radiotherapy at 60–64 Gy in 30–32 fractions, concurrently with two cycles of 500 mg/m^2^ pemetrexed, with nedaplatin doses escalating from 60 mg/m^2^ (level 1) to 70 mg/m^2^ (level 2) and 80 mg/m^2^ (level 3). Consolidation consisted of three pemetrexed/nedaplatin (500 mg/m^2^, 60 mg/m^2^) cycles every 3 weeks after concurrent therapy. The primary objective of the safety was to determine the maximum-tolerated dose (MTD). The secondary endpoints included response rate, PFS and OS.

**Results:**

Fifteen patients were enrolled, including 3, 6 and 6 individuals in the first, second, and third dose levels, respectively. Three cases of dose-limiting toxicities (grade 3 hepatitis, pneumonitis, and grade 4 thrombocytopenia), including one and two patients at levels 2 and 3, respectively, were observed and resulted in discontinued/delayed treatment. Response rates were 86.7 % (95 % confidence interval [CI], 64.2–97.8 %) and 64.3 % (95 % CI, 38.3–85.4 %) at chemoradiation and treatment completions, respectively. Median OS was 30.0 months (95 % CI, 16.4–43.6 months); 2-year OS was 44.0 % (95 % CI, 18.7–69.2 %). Median PFS was 12.0 months (95 % CI, 6.9–17.0 months), and the 2-year PFS 27.0 % (95 % CI, 4.7–49.3 %).

**Conclusions:**

Full dose 500 mg/m^2^ of pemetrexed and nedaplatin 70 mg/m^2^ could be used safely with thoracic intensity-modulated radiotherapy for inoperable stage III lung adenocarcinoma. Further evaluation of stage III lung adenocarcinoma management is warranted.

**Trial registration:**

This study was retrospectively registered at Chinese Clinical Trial Registry (ChiCTR-OPN-16008316, April 2016).

## Background

Approximately one third of all cancer-related deaths are due to lung cancer, which accounts for more deaths than breast, prostate, and colon cancer combined [1]. Non-small-cell lung cancer (NSCLC) accounts for approximately 80 % of all cases of lung cancer [2]. Adenocarcinoma and squamous cell carcinoma are the two major subtypes of non-small cell lung carcinoma, with ~70 % of non-small cell lung carcinoma cases detected at an unresectable stage [3]. Lung adenocarcinoma is the most common type in females (smokers or non-smokers) and non-smoking males; its percentage is higher in Asia than in North America [4]. Concurrent chemoradiotherapy is considered the standard of care for patients with inoperable stages II and III disease [5, 6]. Full-dose chemotherapy with concurrent chemoradiotherapy using a platinum-based third-generation (i.e. paclitaxel, vinorelbine, and docetaxel) doublet results in unacceptable toxicity [7]. Further studies to evaluate potential new chemotherapeutic agents that have radiosensitizing potential to pair with concurrent radiation, and can be used at full dose with thoracic radiotherapy (TRT) for the treatment of locally advanced non-small-cell lung cancer (LA-NSCLC) are necessary to improve efficacy [8]. Pemetrexed, an antifolate that inhibits multiple enzymes (thymidylate synthase, dihydrofolate reductase and glycinamide ribonucleotide formyl transferase) involved in purine and pyrimidine synthesis, has become a drug of choice for patients with lung adenocarcinoma [9, 10]. Nedaplatin is a second-generation platinum derivative, which produces similar antitumor activities, but causes less nausea/vomiting and nephrotoxicity compared with cisplatin [11–14]. IMRT is an effective technique with acceptable acute toxicity, also when (sequentially or concomitantly) combined with chemotherapy [15]. Using IMRT to treat NSCLC leads to low rates of pulmonary and esophageal toxicity, and favorable clinical outcomes in terms of survival [16]. The combination of carboplatin/cisplatin, pemetrexed, and TRT may not be the optimal regimen for locally advanced patients. Therefore, a phase I study was designed to assess the feasibility of a combination of concurrent nedaplatin, pemetrexed and thoracic intensity-modulated, followed by nedaplatin/pemetrexed consolidation without the dose limiting toxicity (DLT) exceeding 33 % in patients with inoperable Stage IIIA/B lung adenocarcinoma.

## Methods

### Patient eligibility

Patients with histologically or cytologically confirmed adenocarcinoma stage IIIA/IIIB, deemed unresectable by the Lung Cancer International Staging System, were eligible. Each patient was a candidate for definitive radiotherapy. Other eligibility criteria included the following: measurable or assessable disease as defined by the response evaluation criteria in solid tumours (RECIST) criteria, performance status (PS) of 0 or 1, absolute neutrophil ≥2000 cells/μL, and platelet count ≥100,000/μL; hemoglobin level ≥9 g/dL; calculated creatinine clearance ≥60 mL/min; bilirubin level ≤2.0 mg/dL; transaminase less than or equal to twice the upper limit of the normal value; forced expiratory volume in 1 s >1.0 L. Exclusion criteria comprised previous surgery, radiation or chemotherapy; >5 % weight loss; clinically significant medical or psychiatric disorders. Bone scan and computed tomographic (CT) scans/magnetic resonance imaging of the chest, abdomen, and brain were performed. All patients provided written informed consent and the study was approved by the local ethics review board. This study was retrospectively registered at Chinese Clinical Trial Registry (ChiCTR-OPN-16008316, April 2016) after patient enrollment.

### Treatment

The research protocol was approved by the ethics committee of the Nanhai Hospital of Southern Medical University. We also obtained the written consent of patients for participation. This study was designed to determine the possibility of administering concomitant pemetrexed/nedaplatin chemotherapy and intensity-modulated radiotherapy followed by pemetrexed/nedaplatin consolidation chemotherapy for inoperable stage III lung adenocarcinoma without the DLT exceeding 33 % in the patients. The secondary objectives included toxicity evaluation of concurrent chemoradiation, consolidation treatment, and complete treatment; assessment of response rate following concomitant treatment and at treatment completion; determination of overall and progression-free survival rates.

For chemotherapy, 500 mg/m^2^ Pemetrexed was administered i.v. on Days 1 and 22 in 250 mL normal saline throughout levels 1–3, with premedication consisting of dexamethasone, folic acid and vitamin B12. Nedaplatin was administered by intravenous infusion for two concurrent cycles every 3 weeks. The dose of nedaplatin was escalated as follows: 60 mg/m^2^ (level 1), 70 mg/m^2^ (level 2), and 80 mg/m^2^ (level 3). Consolidation treatment consisted of three additional cycles of pemetrexed/nedaplatin (500 mg/m^2^, 60 mg/m^2^) every 3 weeks after concurrent therapy.

In the case of radiotherapy, IMRT (intensity-modulated radiotherapy) was delivered to a cumulative dose of 60–64 Gy at 2.0 Gy/fraction. Treatment planning was based on CT simulation. The gross target volume (GTV) included the primary tumor and involved lymph nodes. Involved field irradiation, omitting elective irradiation of lymph nodes, was used in order to optimize definitive dosing to the tumor [17]. A clinical target volume (CTV) was defined around the GTV and subclinical lymph node regions using an expansion of 0.5–1.0 cm for the presumed microscopic extension. A 0.7–0.8 cm margin was added to create an internal target volume (ITV). The planning target volume (PTV) consisted of ITV with the vertical field margins extended to 0.5–1.0 cm and lateral field margins extended to 0.5 cm for setup variations. Normal tissue constraints were as follows: the maximum point dose to the spinal cord, 48 Gy; total lung, V5 < 65 % and V20 < 35 %; mean lung dose, ≤20 Gy; heart, V30 < 50 %. IMRT plans were developed by using a commercial treatment-planning system (XIO-Release 4.80, Elekta, Ltd., Stockholm, Sweden).

### Toxicity assessment and response

Toxicity was assessed using the National Cancer Institute (NCI)’s Common Terminology Criteria for Adverse Events (CTCAE).v4.03 [18]. Assessment of disease response was carried out using the RECIST 1.1 criteria [19, 20] at 3 weeks after chemoradiation completion and a month after consolidation therapy. The best overall response is based on all tumor assessments starting from chemoradiation. Then, restaging scans were performed every 3 months for 1 year, and every 6 months from treatment end.

At least three patients were enrolled for each dose level, and had to have completed concurrent administration of nedaplatin/pemetrexed/radiotherapy without Dose-limiting toxicities (DLT) before escalation to the next dose. If 1 patient experienced DLT, 3 additional patients were accrued. If no more than 1 of the 6 patients experienced DLT, the next three patients were treated at the next higher dose level. If 2 out of 6 patients at a dose level experienced DLT, this level was considered the maximum tolerated dose (MTD). DLT were assessed during chemoradiation and up to 5 weeks after consolidation completion. Dose-limiting toxicities were defined from early and late toxicities as follows: Grade 3/4 hematological toxicity, febrile neutropenia, esophagitis, pneumonitis, or persistent elevation of creatinine, bilirubin and transaminase resulting in preventing treatment, or dosing delay because of toxicity (radiation therapy was delayed by a week or more; the following chemotherapy was delayed by 2 weeks or more, while consolidation therapy was delayed by 4 weeks or more after radiotherapy completion), or late high-grade (>3) bronchopulmonary and esophageal toxicities according to criteria of the Radiation Therapy Oncology Group (RTOG).

### Statistical analyses

Quantitative variables were described as median and standard deviation (SD). A potential follow-up of at least 24 months was required for analysis. Survival was defined as the time from the first day of treatment to death or last follow-up. Progression-free survival was measured from the first day of treatment to the time of disease progression. Overall and progression-free survival rates were estimated using the Kaplan-Meier method [21]. xStatistical analyses were performed using SPSS for Windows version 16.0.

## Results

Between January 2012 and September 2013, 15 patients were enrolled in this study at the Nanhai Hospital of Southern Medical University and the Cancer Center of Guangzhou Medical University. The demographic characteristics of patients are shown in Table 1.

**Table 1:** Patient characteristics

Characteristics	Level 1 (*N* = 3)	Level 2 (*N* = 6)	Level 3 (*N* = 6)	N (%)
Age, y				
Median (range)	59 (65–56)	61 (48–63)	63 (52–68)	62 (48–68)
Gender, n				
Male	1	4	3	8 (53 %)
Female	2	2	3	7 (47 %)
ECOG performance status, n				
0	2	2	2	6 (40 %)
1	1	4	4	9 (60 %)
Clinical stage				
IIIA	1	2	2	5 (33 %)
IIIB	2	4	4	10 (67 %)
Smoking History, n				
Never	2	2	3	7 (47 %)
Ever	1	4	3	8 (53 %)
Former	1	2	1	4 (27 %)
Current	0	2	2	4 (53 %)

At the Level 1 dose, 1 of the first 3 patients experienced grade 3 esophagitis and grade 2 neutropenia during the second cycle; because no DLT was observed at Level 1, nedaplatin dose was escalated to Level 2. One patient receiving Level 2 dose, with hepatitis B virus, developed viral hepatitis that resulted in grade 3 transaminase, and discontinued consolidation therapy after 2 concurrent chemotherapy cycles and full-dose radiation. More than 30 % of patients developed grade 3/4 neutropenia on Dose Level 3. One patient developed grade 4 thrombocytopenia and another experienced grade 3 pneumonitis that lasted at least a week, which were considered DLTs. Esophagus toxicity other than hematological toxicity was well tolerated. All patients completed irradiation (60–64Gy) as prescribed (Table 2). Delay of scheduled radiation therapy owing to esophagitis and pneumonitis occurred in only 2 patients (3 days and 1 week, respectively). The dose-volume histogram showed that the V20 and mean lung dose (MLD) of these patients were 18–33 % and 926–1535 cGy, respectively. A total of 14 patients received consolidation therapy as planned. Table 3 summarizes the grade 3/4 adverse events observed during the chemoradiation and consolidation phases of the study. Severe late toxicities (radiation pneumonitis, prolonged esophagitis, or spinal cord toxicities) were uncommon in all longterm survivors.

**Table 2:** Radiotherapy delivery

Dose level	N	Radiation therapy dose (Gy)			Dose delay due to AE
Level 1	3	62	62^a^	64	1 (3 days)
Level 2	6	64	60	62	0
		64	62	60	
Level 3	6	60	64	62	1 (7 days)
		64	60^b^	62	

^a^Grade 3 esophagitis

^b^Grade 3 pneumonitis

**Table 3:** Grade 3–4 adverse events (CTCAE version 3.0, Concurrent chemoradiotherapy course *N* = 15, Consolidation chemotherapy course *N* = 14)

Toxicity grade 3–4	Concurrent chemoradiotherapy N (%)	Consolidation chemotherapy N (%)
Neutropenia	5 (33.3)	2 (14.3)
Anemia	2 (13.3)	3 (21.4)
Thrombocytopenia	2 (13.3)	1 (7.1)
Febrile neutropenia	1 (6.7)	0 (0.0)
Vomiting	2 (13.3)	0 (0.0)
Esophagitis	3 (20.0)	1 (7.1)
Transaminase	1 (6.7)	0 (0.0)
Pneumonitis	1 (6.7)	0 (0.0)
Creatinine	0	0 (0.0)

**Fig. 1:**
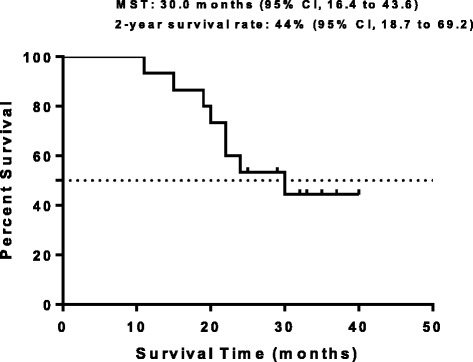
Overall survival curve. MST, median survival time (*N* = 15)

**Fig. 2:**
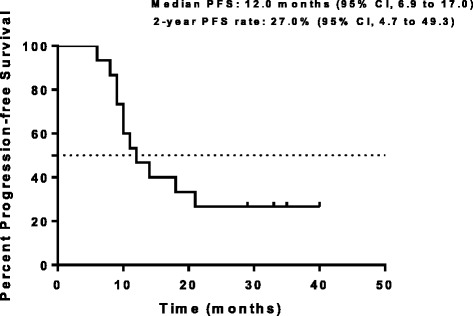
Progression-free survival (PFS) curve (*N* = 15)

In this study, response rates were 86.7 % (95 % confidence interval [CI], 64.2–97.8 %) and 80.0 % (95 % CI, 56.0–94.6 %) at chemoradiation end and treatment completion, respectively (Table 4). The median follow-up time for the censored cases was 26.3 months. The median OS was 30.0 months (95 % CI, 16.4 to 43.6 months), with a 3-year OS rate of 44.0 % (95 % CI, 18.7 to 69.2 %). The median PFS was 12.0 months (95 % CI, 6.9 to 17.0 months), with a 2-year PFS rate of 27.0 % (95 % CI, 4.7 to 49.3 %) (Figs. 1, 2). Only 2 patients had PD, which resulted from liver and contralateral lung metastases. DLTs were observed in one of six patients at level 2, and two of six at level 3. The DLTs observed were grade 3 hepatitis and pneumonitis, and grade 4 thrombocytopenia. Dose-related grade 3 esophagitis, neutropenia, and vomiting were observed but were not dose-limiting. There was no late toxicity greater than grade 3. The MTD was determined to be level 3.

**Table 4:** Efficacy (RECIST version 1.1)

Study phase	Response	N	%
Chemoradiation	CR	0	0.0
	PR	13	86.7
	SD	2	13.3
	PD	0	0.0
Treatment completion	CR	1	6.7
	PR	11	73.3
	SD	3	20.0
	PD	0	0

*CR* complete response, *PR* partial response, *SD* stable disease, *PD* progressive disease

## Discussion

A total of 30–40 % of NSCLC patients present with locally or regionally advanced unresectable tumors. But the optimal regimen, dosage, and administered agents for locally-advanced non-small-cell lung cancer remain controversial. Radiation therapy (RT) combined with chemotherapy is more effective than RT alone, and concomitant chemoradiation has yielded improved survival compared to sequential chemotherapy and RT, but at the cost of heightened toxicity, especially esophagitis [22]. Current international guidelines recommend the use of platinum-based chemotherapy and concurrent thoracic radiotherapy (TRT) for patients with locally advanced non-small-cell lung cancer (NSCLC). Pemetrexed is considered to be less toxic than other cytotoxic agent. Previous studies have demonstrated that full dose pemetrexed-based chemotherapy concurrently with thoracic radiation therapy is feasible for NSCLC patients with unresectable stage III disease [23, 24]. We assessed the optimal dose, toxicity, and tolerability of concurrent dose-escalation of nedaplatin/pemetrexed with TRT followed by consolidation in patients with unresectable locally advanced lung adenocarcinoma.

Nedaplatin, a second-generation cisplatin analog and useful chemotherapeutic agent with radiosensitizing properties, has been developed to reduce cisplatin-induced toxicities, especially in patients with NSCLC, esophageal cancer, uterine cervical cancer, head and neck cancer, or urothelial cancer [25]. It was approved for the treatment of NSCLC, including adenocarcinoma and squamous carcinoma, in China. Teramoto et al. [12] reported a phase II study of docetaxel plus nedaplatin in patients with metastatic non-small-cell lung cancer. They found an overall response rate of 50.0 %; median survival and median progression-free survival times were 13.0 and 7.4 months, respectively. These findings indicate that the docetaxel and nedaplatin combination is well tolerated with potent activity in patients with metastatic NSCLC. Other phase II studies assessed nedaplatin in combination with irinotecan, gemcitabine and paclitaxel, respectively, used as first-line chemotherapy, and reported response rates of 65.8 %, 45.7 % and 53.2 %, respectively, in patients with NSCLC [26–28]. In unresectable stage IIIA or IIIB NSCLC indicated for curative radiotherapy, Sekine et al. [29] reported a phase I study of nedaplatin at 80 mg/m^2^ and escalating doses of paclitaxel from 120 to 150 mg/m^2^ concurrently with thoracic radiotherapy (TRT) in 18 patients. It was concluded that paclitaxel and nedaplatin doses could not be escalated due to severe pulmonary toxicity at level 1. Another phase I/II trial of weekly paclitaxel (35 mg/m^2^) and nedaplatin (20 mg/m^2^) for 6 weeks revealed that this regimen is safe and effective for NSCLC with concurrent TRT [30]. A phase II study led by Oshita et al. [31] evaluated a dose of nedaplatin at 50 mg/m^2^, and irinotecan at 60 mg/m^2^ on days 1 and 8 every 4 weeks for 2–4 cycles with concurrent TRT (2 Gy per day, totaling 60 Gy). This treatment was effective and safe for patients, and 5-year disease-free and overall survival rates were 25.7 % and 40.0 %, respectively. However, no Phase III study assessing chemotherapy or concurrent chemoradiotherapy using nedaplatin has been reported, because nedaplatin is not commonly used throughout the world.

Pemetrexed, a novel, multi-targeted antifolate chemotherapy agent that inhibits target enzymes (thymidylate synthase, dihydrofolate reductase, and glycinamide ribonucleotide formyl transferase), was initially approved for second-line treatment of advanced NSCLC [32]. Pemetrexed was subsequently approved as first-line in advanced non-squamous NSCLC based on a phase III trial showing a survival advantage for pemetrexed–cisplatin compared to gemcitabine–cisplatin [33]. Pemetrexed also is a feasible agent for concurrent chemoradiotherapy and consolidation therapy [34, 35].

Intensity-modulated radiotherapy (IMRT) is an advanced radiotherapy that uses intensity-modulated beams, which can provide multiple intensity levels for any single beam direction and a given source position, allowing shaped distributions and dose gradients with narrower margins than previously possible [36]. In comparison with 3D-CRT, involved-field radiotherapy (IF-RT) and IMRT combination leads to a significantly better sparing of normal tissues and higher total doses, while the potential therapeutic drawback of decreased incidental irradiation of elective lymph nodes is moderate [37].

Both agents have demonstrated safety and efficacy in locally advanced non-small cell lung cancer in conjunction with Radiation therapy.

On the definition of Dose-limiting toxicities, there are some difference among them because of the various methods and theories. In most cases, DLTs were defined as severe toxicity leading to dose reduction or treatment discontinuation [38–40]. According to the criteria for DLT in the protocol, we determine The MTD to be level 3 (nedaplatin 80 mg/m^2^) in this trial. The recommended dose is to be the best tolerated dose immediately below the MTD [41]. In the majority of the trials, the recommended dose was usually defined as one dose level below the MTD. But in some trials the recommended dose was equivalent to the MTD. There was lack of standardization, so we recommend the lower dose for safety. This study identified the recommended nedaplatin dose at 70 mg/m^2^ for phase II evaluation. The dose of chemotherapy in current study was lower than that in a Japanese phase III study of 100 mg/m^2^ nedaplatin and 60 mg/m^2^ docetaxel, or 80 mg/m^2^ cisplatin and 60 mg/m^2^ docetaxel for squamous cell lung cancer with stage IIIB/IV or postoperative recurrence [42]. They showed that OS (13.6 vs 11.4 months) was significantly longer in the nedaplatin group, but grade 3 or worse leucopenia (55 % vs 45 %), neutropenia (82 % vs 71 %), and thrombocytopenia (9 % vs 0 %) were more frequent in the nedaplatin group. Toxicity were more serious compare to the outcome of this study. To avoid severe adverse effects and interruption of radiationtherapy, appropriate dose reduction would be feasible. Indeed, the comparatively small sample size and short follow-up time in the current investigation present limitations. Future studies are warranted.

The response group was defined as patients achieving a CR or PR. In the chemoradiation phase and treatment completion, the response rate was 86.7 % and 80.0 %, respectively. The majority of patients experienced PR (thirteen patients) and two patients had SD in chemoradiation phase. At completion of treatment three patients had SD, in addition, one patient obtained CR and thirteen patients PR as their best overall tumour response. Further more, two of the three patients had SD for at least 6 months. The median OS was 30.0 months with a 3-year OS rate of 44.0 %. The median PFS was 12.0 months with a 2-year PFS rate of 27.0 %. Our results are consistent with previous studies. A phase I study of pemetrexed plus cisplatin followed by pemetrexed consolidation therapy with dose-escalation of TRT in patients with locally advanced nonsquamous NSCLC showed that the objective response rate was 83 %. A phase II study of pemetrexed plus cisplatin with concurrent TRT in Stage IIIA or Stage IIIB non-squamous NSCLC showed a best overall response of 72 %(PFS, 13.8 months; OS, 26.2 months) [43]. Furthermore, a randomized phase III study was performed to investigate the effect of pemetrexed 500 mg/m^2^ + cisplatin 75 mg/m^2^ or etoposide 50 mg/m^2^ + cisplatin 50 mg/m^2^ with plus concurrent TRT followed by pemetrexed consolidation cytotoxic chemotherapy in locally advanced nonsquamous NSCLC [44]. It shows that median PFS, ORR and disease control rate was respectively 11.4 months, 35.9 % and 80.7 % in the pemetrexed-cisplatin group and 9.8 months, 33.0 % and 70.7 % in the etoposide-cisplatin group. The Pem + Cis arm did not improve PFS compared with the control arm, but had a greater security.

## Conclusions

In conclusion, the present phase I study is the first of its kind assessing combination therapy by nedaplatin and pemetrexed with thoracic intensity-modulated radiotherapy for inoperable stage III lung adenocarcinoma. The DLTs seen with this combination were hepatitis, thrombocytopenia, febrile neutropenia, and pulmonary toxicity. Preliminary local disease control and overall survival are encouraging. Our findings suggest that full dose 500 mg/m^2^ of pemetrexed and nedaplatin 70 mg/m^2^ could be used safely with thoracic intensity-modulated radiotherapy for inoperable stage III lung adenocarcinoma.. The response rate, PFS, and overall survival are encouraging. An ongoing phase II/III study is to evaluate the efficacy of the same chemoradiation platform as the present trial or cisplatin and pemetrexed in patients with unresectable stage III lung adenocarcinoma. Further evaluation of stage III lung adenocarcinoma management is required.

## Abbreviations

CI: Confidence interval
CR: Complete response
CT: Computed tomography
CTCAE: National cancer institute (NCI)’s common terminology criteria for adverse events
CTV: Clinical target volume
DLT: Dose limiting toxicity
GTV: Gross target volume
IF-RT: Involved-field radiotherapy
ITV: Internal target volume
LA-NSCLC: Locally advanced non-small-cell lung cancer
MRI: Magnetic resonance imaging
MST: Median survival time
MTD: Maximum-tolerated dose
NSCLC: Non-small-cell lung cancer
OS: Overall survival
PD: Progressive disease
PFS: Progression-free survival
PR: Partial response
PS: Performance status
PTV: Planning target volume
RECIST: Response evaluation criteria in solid tumors
RT: Radiation therapy
RTOG: Radiation therapy oncology group
SD: Stable disease
TRT: Thoracic radiotherapy
